# Cerebrovascular events in hemodialysis patients; a retrospective observational study

**DOI:** 10.1186/s12882-019-1629-y

**Published:** 2019-12-12

**Authors:** Ruya Ozelsancak, Hasan Micozkadioglu, Dilek Torun, Nihan Tekkarismaz

**Affiliations:** Department of Nephrology, Adana Turgut Noyan Teaching and Research Center, Baskent University School of Medicine, Dadaloglu Mah, 39/6, Yuregir, 01250 Adana, PK Turkey

**Keywords:** Cerebrovascular event, Cerebral small vessel disease, Hemodialysis, Hemorrhage, Ischemia, Stroke

## Abstract

**Background:**

This study reports findings in subjects who underwent brain imaging for any reason, and examined factors influencing cerebrovascular events (CVEs) in hemodialysis (HD) patients.

**Methods:**

We reviewed the files of patients on HD between January 2015 and January 2018. A total of 432 patients who underwent HD for at least 5 months by the January 2015 and who were older than 18 years were included in the study; 264 had been examined by cerebral computed tomography or magnetic resonance imaging examination within the 3 years. Cerebrovascular pathology was detected in 139 of 264 patients.

**Results:**

Of the 139 patients, 65 (24.62%) had ischemic lesions, 25 (9.47%) had hemorrhagic lesions, and 49 (18.56%) had cerebral small vessel disease (CSVD). We compared recorded data and later clinical findings between patients with and those without CVEs. The cause of end-stage renal disease was diabetes in 58.5% of patients with ischemic lesions, 52% in those with hemorrhagic lesions, and 55% in those with CSVD (*P* < 0.05). Patients with cerebrovascular ischemia were older (*P* = 0.0001) and had lower serum creatinine (sCr) (*P* = 0.0001) and higher serum C-reactive protein (CRP) (*P* = 0.002) levels than normal subjects. Hemorrhagic patients were older (*P* = 0.003) and had lower sCr (*P* = 0.003) and serum predialysis potassium (*P* = 0.003) and parathyroid hormone (PTH) (*P* = 0.004) levels than normal subjects. Patients with CSVD were older (*P* < 0.0001) and had lower sCr (*P* < 0.0001), phosphorus (*P* < 0.007), and PTH (*P* < 0.013) and higher CRP (*P* < 0.002) levels than normal subjects.

**Conclusions:**

HD patients with CVEs are older and typically have diabetes mellitus and lower sCr levels.

## Background

Stroke is a major cause of morbidity and mortality worldwide and is a public health burden. Risk factors for stroke are similar to those for cardiac and peripheral vascular disease; non-modifiable risk factors include older age, male sex, diabetes, and family history. Diabetes mellitus (DM), hyperlipidemia, and smoking are risk factors for atherosclerosis and increase the risk of ischemic stroke; hypertension is the major modifiable risk factor for both ischemic and hemorrhagic strokes; and bleeding diathesis and blood vessel wall fragility increase susceptibility to hemorrhagic stroke [1, 2].

Cerebral small vessel disease (CSVD) is a syndrome characterized by perforation of cerebral arterioles, capillaries, and venules and imaging changes in white matter and subcortical grey matter such as small subcortical infarction, lacunes, white matter hyperintensities, prominent perivascular spaces, cerebral microbleeding, and atrophy. CSVD leads to dementia and stroke in up to 45 and 20% of cases, respectively [3].

Patients with end-stage renal disease (ESRD) have advanced atherosclerosis of the cerebral vasculature compared with the general population and require dialysis therapy, which is associated with increased risk of ischemic or hemorrhagic stroke due to the interaction between vascular comorbidities linked to kidney impairment and pathologies resulting from uremia such as vascular calcification and malnutrition-inflammation-atherosclerosis syndrome [4, 5]. ESRD is also associated with hypertension and bleeding diathesis, and routine administration of heparin during hemodialysis (HD) can increase the risk of hemorrhagic stroke.

Previous studies in the United States and Japan have reported 2- to 10-fold increased risk of stroke in dialysis patients compared with the general population, with the risk attributable to ESRD being greatest for older subjects and females in the United States [6–8]. Older age, male sex, diabetes, and hypertension were found to be risk factors for both ischemic and hemorrhagic strokes in a Japanese study [8].

Previous studies have shown that HD patients are more susceptible to stroke; however, these investigations have focused on overall risk and have relied on International Classification of Diseases (ICD) codes, while some patients may exhibit cerebrovascular changes without progressing to stroke. The aim of the present study was to examine patients who had undergone brain imaging for any reason (imbalance, dizziness, numbness, or paralysis) and identify factors affecting cerebrovascular events (CVEs) in HD patients.

## Methods

### Patients

This retrospective observational study was approved by the Clinical Research Ethics Committee of the Faculty of Medicine, Baskent University.

The files of patients who were on HD between January 2015 and January 2018 were reviewed. We included 432 patients who had undergone HD for at least 5 months by the January 2015 and who were older than 18 years. Duration of dialysis was calculated in months from start of dialysis to january 2015. Patient age, sex, dialysis duration, and comorbidities such as hypertension, DM, coronary artery disease (CAD), and renal failure were recorded. Patients who had a history of cancer or who had experienced stroke before dialysis therapy were excluded. We also recorded laboratory results (mean values) of the previous 5 months at the start of the study.

Patients who were previously diagnosed with type 1 or 2 diabetes were considered to have DM. If they had blood pressure higher than 140/90 mmHg on two separate occasions or were taking anti-hypertensive medication were considered hypertensive. A history of angina pectoris, angioplasty, myocardial infarction, or congestive heart failure was defined as CAD. We also recorded laboratory parameters such as urea, creatinine (Cr), potassium, sodium, phosphorus, serum albumin, Kt/V (where K is dialyzer clearance of urea; t is dialysis time; and V is volume of distribution of urea), C-reactive protein (CRP), uric acid, hemoglobin, triglyceride, total cholesterol, low-density lipoprotein cholesterol, high-density lipoprotein cholesterol levels in blood samples drawn before the second HD session of the week.

Data for patients who underwent brain imaging [magnetic resonance imaging (MRI) or computed tomography (CT)] for any reason (imbalance, dizziness, numbness, or paralysis) were evaluated.

### Statistical analysis

Statistical analyses were performed using IBM SPSS Statistics for Windows v.17.0 (SPSS Inc., Chicago, IL, USA). Continuous variables with normal distribution are presented as the mean (standard deviation), and non-normally distributed variables are presented as the median (range). Comparisons between two groups were carried out with the Student’s *t*-test and Mann-Whitney U test for normally and non-normally distributed data, respectively. Categorical variables were compared between groups with the chi-square test or Fisher’s exact test. *P* < 0.05 was considered statistically significant. Then, the significant variables from the univariate model were placed in a logistic regression analysis to further analyze possible risk factors (entry: *P* ≤ 0.05; removal: *P* > 0.1).

## Results

**Fig. 1:**
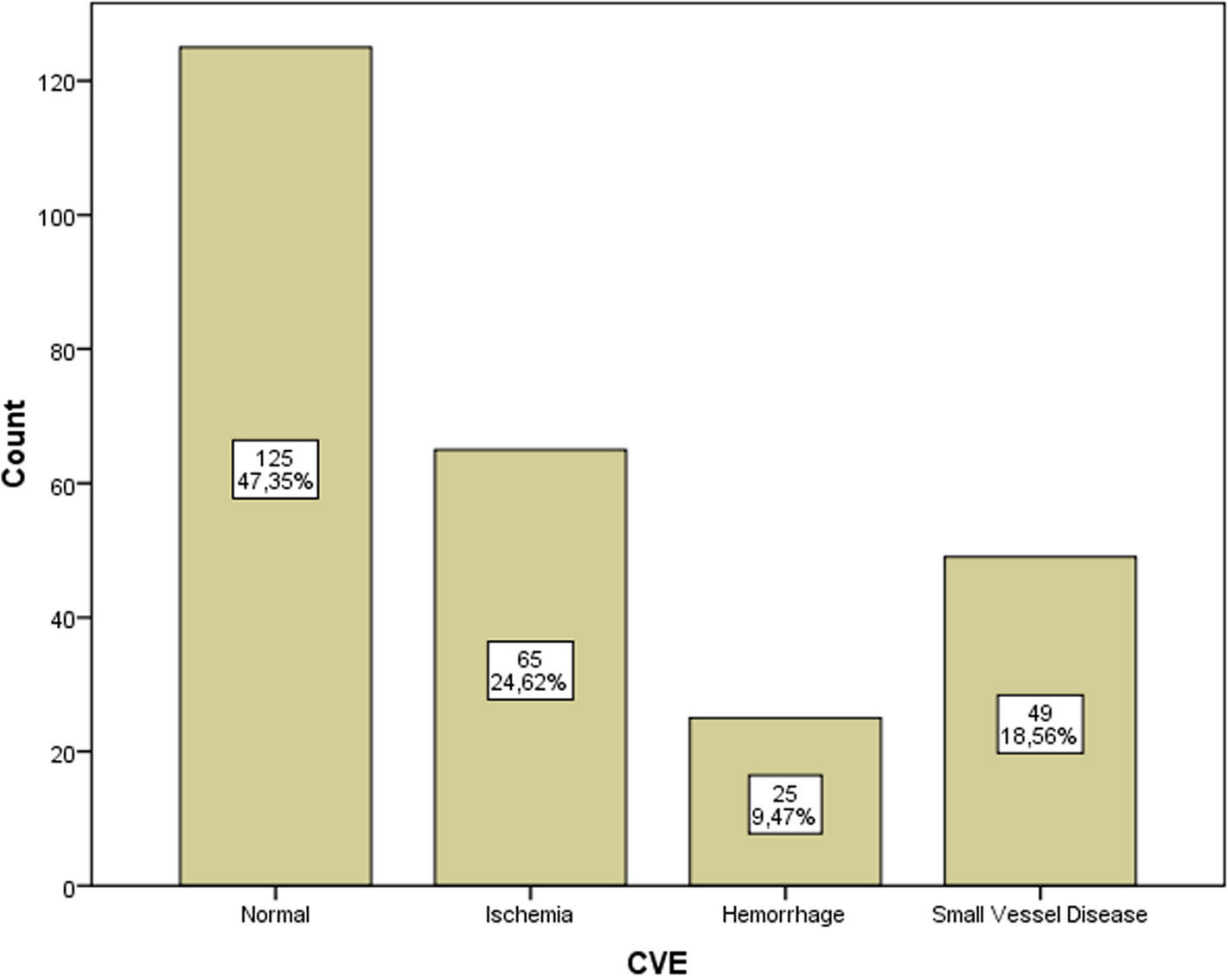
Results of cerebrovascular imaging

Over the 3 years of observation, 264 patients had cerebral CT or MRI. Causes and types of radiological imaging studies have shown in Table 1. We compared the initially recorded data and subsequent clinical findings of the patients who had cerebrovascular pathology (in 139 patients, 52.6%) and who had not. Of these, 65 (24.62%) had ischemic lesions, 25 (9.47%) had hemorrhagic lesions, and 49 (18.56%) had CSVD Fig. 1. The mean ages of patients in different subgroups were as follows: normal imaging findings, 51 ± 15.8 years; ischemia, 61.6 ± 13.8 years; hemorrhage, 61 ± 14 years; and CSVD, 67 ± 10 years, with no statistically significant difference between them (Table 2). The initially recorded data and subsequent clinical findings of patients with and without CVE are compared in Table 3.

**Table 1:** Causes and types of radiological imaging studies

	Ischemia n (%)	Hemorrhage n (%)	Small vessel disease n (%)	Normal imaging n (%)
Change of consciousness	16 (25)	8(32)	16 (33)	19 (15)
Imbalance, ataxia, paralysis, numbness	16 (25)	11 (44)	10 (20)	31 (25)
Headache	9 (14)	4 (16)	9(18,4)	38 (30)
Dizziness	10 (15)	–	5 (10,2)	17 (14)
Other: dysarthria, tinnitus, convulsions	14 (21)	2 (8)	9 (18,4)	20 (16)
Type of imaging				
CT	25 (38)	23 (92)	25 (51)	88 (70)
CT + MRI	26 (40)	–	17 (35)	7 (6)
MRI	14 (22)	2 (8)	7 (14)	30 (24)

Abbreviations: *CT* Computed tomography; *MRI* Magnetic resonance imaging

**Table 2:** Baseline demographic and clinical characteristics of patients

	Normal imaging (*n* = 125, 47.3%)	Ischemia (*n* = 65, 24.62%)	Hemorrhage (*n* = 25, 9.47%)	CSVD (*n* = 49, 18.56%)
Age (years)	51 ± 15.8	61.6 ± 13.8	61 ± 14	67 ± 10
Sex (F,%)	57.6	63.1	52	44.9
Time on dialysis (months)	43 (5–200)	56 (6–240)	48 (5–216)	34 (6–305)
ESRD etiology				
DM, n (%)	41 (32,8)	38 (58.5)*	13 (52)*	27 (55)*
HT, n (%)	5 (4)	4 (6,2)	5 (20)*	5 (10)
GN, n (%)	11 (8.8)	2 (3.1)	0 (0)	1 (2)
PKD, n (%)	5 (4)	2 (3.1)	0 (0)	3 (6.1)
VUR, n (%)	1 (0.8)	0 (0)	1 (4)	0 (0)
Other, n (%)	61 (48.8)	18 (27.7)	6 (24)	13 (26.5)
Unknown, n (%)	1 (0.8)	1 (1.5)	0 (0)	0 (0)
CAD, n (%)	36 (28)	39 (60)*	12 (48)*	30 (61)*

Abbreviations: *CAD* coronary artery disease; *CSVD* cerebral small vessel disease; *DM* diabetes mellitus; *ESRD* end-stage renal disease; *GN* glomerulonephritis; *HT* hypertension; *PKD* polycystic kidney disease; *VUR* vesicoureteral reflux

*P ≤ 0.005

**Table 3:** Laboratory values of patients

	Normal imaging (*n* = 125, 47.35%)	Ischemia (*n* = 65, 24.6%)	Hemorrhage (*n* = 25, 9.47%)	Small vessel disease (*n* = 49, 18.56%)
Creatinine (mg/dL)	9.09 ± 2.4	8.06 ± 2.6*	7.3 ± 2.5*	8.51 ± 7.06*
Potassium (mEq/l)	5.08 ± 0.61	5.11 ± 0.68	4.62 ± 0.68*	5.10 ± 0.87
Phosphorus (mg/dL)	5 ± 1.36	4.87 ± 1.28	4.72 ± 1.54	4.30 ± 1.57*
Triglyceride (mg/dl)	193.8 ± 131.6	183.3 ± 120.5	200 ± 145	182 ± 86.5
Total cholesterol (mg/dL)	167.4 ± 44.6	171.9 ± 50.1	177.4 ± 50.7	178.9 ± 44.6
LDL cholesterol (mg/dL)	88.1 ± 37.5	97.2 ± 41.2	94.3 ± 33.7	101.7 ± 35.4
HDL cholesterol (mg/dL)	40.2 ± 13.3	39.6 ± 11.6	43.5 ± 11.7	45.5 ± 29.7
PTH (pq/ml; median, min-max)	427 (1–3145)	353 (34–1900)	174 (10–1212)*	296 (4–1287)*
CRP (mg/L)	4.8 (1–103)	9 (2–68)*	6 (2–56)	8 (2–208)*
Uric acid (mg/dL)	6.99 ± 5.53	6.52 ± 1.25	6.25 ± 1.09	6.61 ± 1.24
Hemoglobin (g/dL)	11.7 ± 1.7	11.2 ± 1.4	10.8 ± 1.3	10.8 ± 1.2
Kt/V	1.46 ± 10.29	1.37 ± 0.24	1.47 ± 0.22	1.45 ± 0.26

Abbreviations: *CRP* C-reactive protein; *CSVD* cerebral small vessel disease; *HDL* high-density lipoprotein; *Kt/V* [dialyzer clearance of urea (K) × dialysis time (t)]/volume of distribution of urea (V); *LDL* low-density lipoprotein; *PTH* parathyroid hormone

*P ≤ 0.005

There were no differences among patients in terms of sex and time on dialysis. The cause of ESRD was diabetes in 32.8% of patients with normal imaging, 58.5% of patients with ischemic lesions, 52% of patients with hemorrhagic lesions, and 55% of patients with small-vessel ischemia (*P* < 0.05; Table 2). The frequency of CAD was higher in patients with CVE than in other subjects (*P* = 0.0001; Table 2).

We compared patients with cerebrovascular ischemia and those with normal cerebrovascular imaging findings and found that the former group was older [61.6 ± 13.8 vs. 51 ± 15.8 years, P = 0.0001] and had lower serum (s)Cr [8.06 ± 2.65 vs. 9.09 ± 2.42 mg/dl, P = 0.0001] and higher serum CRP [9 (2–68) vs. 4.8(1–103) mg/l, *P* = 0.002] levels than the latter.

A comparison of patients with cerebrovascular hemorrhage and normal cerebrovascular imaging findings revealed that hemorrhagic patients were older [61 ± 14 vs. 51 ± 15.8 years, *P* = 0.003] and had lower sCr [7.36 ± 2.52 vs. 9.09 ± 2.42 mg/dl, P = 0.003], predialysis serum potassium [4.62 ± 0.68 vs. 5.08 ± 0.61 mEq/l, P = 0.003], and serum PTH [174 (10–1212) vs. 427 (1–3145) pq/ml, *P* = 0.004] levels than normal subjects.

Finally, we compared patients with CSVD and those with normal cerebrovascular imaging findings. CSVD patients were older [67 ± 10 vs. 51 ± 15.8 years, *P* = 0.0001] and had lower sCr [8.51 ± 7.06 vs. 9.09 ± 2.42 mg/dL, P = 0.0001], serum phosphorus [4.3 ± 1.57 vs. 5.00 ± 1.36 mEq/l, *P* = 0.007], and serum PTH [296 (4–1287) vs. 427 (1–3145) pq/ml, *P* = 0.013] levels and a higher serum CRP [8 (2–208) vs. 4.8 (1–103) mg/l, *P* = 0.002] level than normal subjects. There were no differences in serum lipid levels and Kt/V between the two groups.

The variables which are significant in univariate analysis were evaluated by multivariate analysis. But the result of multivariate analysis were not statistically significant.

## Discussion

The incidence of stroke is 2–10 times higher in ESRD patients on dialysis than in the general population [6–8]. The initiation of dialysis is itself associated with a heightened risk of stroke. Risk factors for stroke in the general population including older age, hypertension, diabetes, obesity, and cigarette smoking also increase the risk of developing chronic kidney disease.

Despite the high prevalence of CVE in the dialysis population, the different subtypes have not been well studied in patients who have had cerebral imaging. We found here that patients with CVE were older and had lower sCr levels than normal subjects. While DM was the primary cause of renal failure, CAD was more frequently observed in CVE patients than in normal subjects. The risk factors for stroke identified in the present study (older age, DM, and CAD) are consistent with previous reports [8–11].

The sCr levels were lower in all subgroups in our study. The sCr originates from creatinine, which is derived from skeletal muscle and is influenced by both muscle mass and glomerular filtration rate; it can serve as a biomarker for various pathological states including ESRD, especially as an indicator of muscle mass. Low muscle mass resulting from protein-energy wasting is associated with poor outcome in HD patients; on the other hand, elevated sCr is linked to longer survival [12, 13]. In the ARNOS study of 1205 patients who were followed up for 1 year, sCr level was a strong predictor of mortality, with a lower survival rate among patients with a predialysis sCr concentration < 8.11 mg/dl [14]. Reduced sCr level reflects a loss of muscle mass or low protein intake, which can lead to adverse outcomes in HD patients.

Cr is a small molecule that is cleared by dialysis; predialysis sCr level may thus be influenced by the dialysis dose in the preceding session, i.e., a higher dose may lower predialysis Cr. We were unable to confirm muscle mass and establish the precise mechanism by which decreased sCr causes CVEs; there have been no studies reporting either a negative or positive association between these two parameters. However, one study investigating the relationship between modified Cr index (which was calculated based on age, sex, predialysis sCr concentration, and Kt/V for urea) for cardiovascular events and all-cause mortality in patients undergoing HD showed that mortality and risk of heart disease were significantly higher in the lower quartiles, while modified Cr index was unrelated to stroke [15]. Thus, the role of sCr concentration in CVE and stroke risk remains unclear.

Many ESRD patients have chronic inflammation. In our study, CRP level was higher in ischemic and CSVD patients. Inflammation contributes to the development of atherosclerosis. In the Rotterdam Scan Study, subjects with elevated CRP levels had a higher frequency of lacunar infarcts than the general population [16]. The LIMIT study showed that compared with the bottom quartile, subjects with CRP in the top quartile were at increased risk of recurrent ischemic stroke, implying that CRP predicts the occurrence of major vascular events [17]. However, these studies were carried out in patients with normal renal function. It has been reported that HD patients show a higher rate of silent cerebral infarctions, which is reflected by elevated CRP [18]. On the other hand, another report found no association between CRP level and risk of cerebrovascular events in HD patients [19].

We observed that predialysis serum potassium concentration was lower in hemorrhagic stroke patients. A study in rats showed that a high potassium diet allowed cerebral arteries to withstand high blood pressure and reduced damage to the artery wall, thereby preventing brain hemorrhage and infarct and lowering mortality rate. Hypertension does not inevitably lead to arterial hypertrophy because it can be alleviated by a high-potassium diet [20, 21]. In a rat model of ischemic stroke, dietary potassium supplementation reduced infarct size in cerebral ischemia by reversing cerebral artery hypertrophy and providing neuroprotection independent of blood pressure, possibly through changes in vascular structure [22]. Given that the primary excretion route of potassium is through the kidney, ESRD patients should be given a potassium-restricted diet as it is difficult to maintain normokalemia during dialysis.

PTH was low in patients with CSVD or in those who experienced hemorrhagic events. Higher PTH levels have been previously observed in stroke patients [23]. In the Atherosclerosis Risk in Communities brain MRI study, a cross-sectional analysis revealed that elevated PTH level was associated with higher white matter hyperintensity score (a sign of CSVD) and more frequent infarcts. White matter hyperintensities of presumed vascular origin are a common finding in brain MRI or CT scans in older subjects and stroke patients, and are associated with a three times higher risk of stroke. However, these authors did not find an association between elevated PTH level and progression of cerebrovascular changes in brain MRI over a 10-year interval [24]. A longitudinal study of HD patients in the Japan Renal Data Registry found that hemorrhagic stroke was associated with PTH concentration > 500 pg/ml, whereas the incidence of ischemic stroke was unrelated to PTH level [25]. The PTH receptor is expressed on endothelial cells, vascular smooth muscle cells, and cardiomyocytes. PTH induces the expression of proinflammatory and atherosclerotic mediators including interleukin-6 and receptor of advanced glycation end products in endothelial cells [26]. The association between PTH and atherogenesis can be explained by vascular calcification and remodeling resulting from the direct interaction of PTH with its receptor on the vessel wall, indirect inflammation, and vascular dysfunction [27]. Most of our patients were taking calcium containing phosphorus binders, which could be the reason for low PTH concentration in those who experienced hemorrhagic events or with CSVD, although the levels were still higher than normal. The use of calcium-containing phosphorus binders can lead to vascular calcification; the resulting impairment in vessel elasticity may cause bleeding in cerebral vessels upon sudden blood pressure changes [28].

## Conclusions

The major findings of this study were the decreased Cr and potassium levels in CVE patients. Although serum PTH, potassium, and phosphorus were lower and CRP was higher in some subjects, we were unable to determine the relationship between these findings and CVEs due to the observational design of the study. On the other hand, we examined all of the cerebral imaging results of patients rather than simply searching stroke ICD codes in the database; we think this was an important point in terms of detecting cerebral changes that do not lead to stroke.

There were several limitations to our study including its observational nature and small sample size, which prevented the establishment of a causal relationship between our observations. We also lacked patient information such as smoking status, body mass index, and physical activity. Additionally, given that we excluded patients with prior events, we were unable to identify recurrent events during the observational period. On the other hand, we did not exclude a small group of patients who had undergone parathyroidectomy as well as HD for ultrafiltration due to heart failure.

## Data Availability

The datasets analyzed during the current study are available from the corresponding author on reasonable request.

## Abbreviations

CAD: Coronary artery disease
CRP: C-reactive protein
CSVD: Cerebral small vessel disease
CT: Computed tomography
CVEs: Cerebrovascular events
DM: Diabetes mellitus
ESRD: End-stage renal disease
HD: Hemodialysis
MRI: Magnetic resonance imaging
PTH: Parathyroid hormone
sCr: Serum creatinine
